# Effect of HIV-exposure and timing of anti-retroviral treatment on immunogenicity of trivalent live-attenuated polio vaccine in infants

**DOI:** 10.1371/journal.pone.0215079

**Published:** 2019-04-19

**Authors:** Shelina Moonsamy, Melinda Suchard, Shabir A. Madhi

**Affiliations:** 1 Centre for Vaccines and Immunology, National Institute for Communicable Diseases, a division of the National Health Laboratory Service, Johannesburg, South Africa; 2 Department of Health Sciences, University of Johannesburg, Johannesburg, South Africa; 3 Chemical Pathology, Faculty of Health Sciences, University of the Witwatersrand, Johannesburg, South Africa; 4 South African Medical Research Council, Respiratory and Meningeal Pathogens Research Unit, Faculty of Health Sciences, University of Witwatersrand, Johannesburg, South Africa; 5 Department of Science and Technology, National Research Foundation, Faculty of Health Sciences, University of Witwatersrand, Johannesburg, South Africa; University of Cape Town, SOUTH AFRICA

## Abstract

**Introduction:**

The prevalence of HIV infection in South African pregnant women has been approximately 30% over the past decade; however, there has been a steady decline in mother-to-child transmission of HIV from 8% in 2008 to <2% in 2015. We evaluated the immunogenicity of live-attenuated trivalent oral polio vaccine (OPV) following the primary vaccination series (doses at birth, 6, 10 and 14 weeks of age) in HIV-exposed uninfected (HEU), HIV-infected infants initiated on early anti-retroviral treatment (HIV+/ART+), HIV-infected infants on deferred ART (HIV+/ART-) and HIV-unexposed infants (HU) as the referent group.

**Methods:**

Serum polio neutralization antibody titres were evaluated to serotype-1, serotype-2 and serotype-3 at 6, 10 and 18 weeks of age. Antibody titres ≥8 were considered seropositive and sero-protective.

**Results:**

At 18 weeks of age, following the complete primary series of four OPV doses, no differences in GMTs, percentage of infants with sero-protective titres and median fold change in antibody titre (18 weeks vs 6 weeks) were observed in HEU infants (n = 114) and HIV+/ART+ infants (n = 162) compared to HU infants (n = 104) for the three polio serotypes. However, comparing HIV+/ART- infants (n = 70) to HU infants at 18 weeks of age, we observed significantly lower GMTs for serotype-1 (p = 0.022), serotype-2 (p<0.001) and serotype-3 (p<0.001), significantly lower percentages of infants with sero-protective titres for the three serotypes (p<0.001), and significantly lower median fold change in antibody titre for serotype-1 (p = 0.048), serotype-2 (p = 0.003) and serotype-3 (p = 0.008).

**Conclusion:**

Delaying initiation of ART in HIV-infected infants was associated with an attenuated immune response to OPV following a four-dose primary series of vaccines, whereas immune responses to OPV in HIV-infected children initiated on ART early in infancy and HEU children were similar to HU infants.

## Introduction

Despite significant advances since the launch of the Global Polio Eradication Initiative in 1988, wild poliovirus and circulating vaccine-derived polioviruses remain a risk. The success of the Global Polio Eradication Initiative thus far is largely attributed to polio vaccination, with the key approach being to maintain vaccine coverage of more than 80% to optimise population immunity [1–3]. South Africa has a high prevalence of human immunodeficiency virus (HIV) in pregnant women, which has remained unchanged at around 30% for over a decade [4]. Although the percentage of infants born to HIV-infected women has remained fairly consistent since 2004, there has been a decline in mother-to-child transmission of HIV from 8% in 2008 to <2% in 2015 through effective perinatal HIV prevention programs, and therefore an increase in HIV-exposed uninfected (HEU) infants [4–6]. It has been demonstrated that HEU infants suffer increased morbidity and mortality than HIV-unexposed (HU) infants, although underlying reasons are unclear [7, 8]. Immunological aberrations, immune system impairment and reduced transfer of maternal antibodies have been reported in HEU infants compared to HU infants [7, 9, 10]. However, immune responses to vaccines in HEU infants has generally been similar to HU infants following routine immunisation schedules [9].

HIV induced immune system impairment is known to negatively influence the immunogenicity and efficacy of vaccines [11], subsequently affecting the vulnerability of an individual to vaccine-preventable diseases [12–14]. As seen for measles, unless severely immunocompromised, an accelerated immunisation schedule is recommended in children who are HIV-infected [15, 16]. The landscape of management of HIV-infected children with anti-retroviral therapy (ART) has changed since the Children with HIV Early Anti-retroviral (CHER) study, which reported better survival in HIV-infected children initiated on ART immediately upon diagnosis of being HIV-infected at 4–8 weeks of age compared to those in whom ART was deferred until clinical or immunological deterioration [17]. The CHER study led to revised recommendations for the management of HIV-infected children in South Africa and globally [18]. However, despite effective ART regimes, reduced immunogenicity and accelerated waning of immunity has been observed in HIV-infected compared to HIV-uninfected children [12, 13, 19–24].

There are limited studies on immune responses to live-attenuated trivalent oral polio vaccine (OPV) in HIV-infected or HEU children [14, 25–27]. Furthermore, there has been no published study comparing immune responses to OPV in HIV-infected infants initiated on early ART compared to those in whom ART is deferred until clinical or immunological HIV disease progression. The aim of our study was to assess the effect of HIV exposure and timing of ART initiation in HIV-infected children on immune responses to a live-attenuated trivalent OPV.

## Materials and methods

### Study population

This study analysed serum samples archived at -70°C at the Respiratory and Meningeal Pathogens Research Unit, Soweto, Johannesburg. Criterion for sample selection was a sample volume of ≥100μl. Briefly, samples were from infants enrolled at 6 to 12 weeks of age from April 2005 to June 2006 in whom the effects of HIV and ART on antibody responses to pneumococcal conjugate vaccine were evaluated [20]. Infants were enrolled into four groups; HU, HEU, HIV-infected infants with CD4^+^ ≥25% randomised to deferred ART (HIV+/ART-) and HIV-infected infants with CD4^+^ ≥25% randomised to immediate ART (HIV+/ART+). The HIV-infected infants were co-enrolled from the CHER study in South Africa [17]. ART was initiated in HIV+/ART- infants when clinically or immunologically indicated, with follow up visits and evaluation every 4 weeks after randomisation until week 24, every 8 weeks thereafter until week 48 and every 12 weeks thenceforth [17]. Clinical criteria for initiation of ART were Centers for Disease Control and Prevention stage C or severe stage B events, and immunologic criteria were CD4^+^ levels of <25% or CD4^+^ counts of less than 1000 cells per cubic millimetre [17]. The first-line ART regimen consisted of zidovudine, lamivudine, and lopinavir-ritonavir given twice daily [17]. Detailed information on these children has been described [13, 17, 20]. The polio vaccination schedule administered during 2005 to 2006 consisted of 6 doses of trivalent OPV, given at birth, 6, 10 and 14 weeks of age, and boosters at 18 months and 6 years [28].

We analyzed for serum polio neutralising antibody titres at 6 weeks of age (i.e. 6 weeks after the birth OPV dose), at 10 weeks of age (i.e. after OPV doses at birth and 6 weeks), and at 18 weeks of age (i.e. following the complete OPV primary schedule of doses at birth, 6, 10 and 14 weeks).

### Laboratory testing

Laboratory testing to determine poliovirus antibody titres by serum antibody neutralisation assays was performed at the Centre for Vaccines and Immunology at the National Institute for Communicable Diseases. Serum samples were heat inactivated at 56°C for thirty minutes to inactivate complement. Samples diluted 1/4 were added to an equal volume of diluent in triplicate to a 96 well plate (wells A1, A2 and A3) and subsequently diluted two-fold serially to obtain a starting dilution of 1/8 up to a final dilution of 1/1024 in wells H1, H2 and H3 (total of 24 wells). An equal volume of a set strength of antigen of poliovirus serotype-1, serotype-2 and serotype-3 (NIBSC Sabin reference strains; 01/528, 01/530 and 01/532 respectively) was added to the sera. Test plates were incubated in a moist 35°C, 5% CO_2_ incubator for 3 hours to allow specific antigen-antibody binding. Following the incubation, a cell suspension of 3 x 10^5^ cells/ml of Hep2C cells (human cervix carcinoma cells of epithelial morphology, supplied by the Centers for Disease Control and Prevention, Atlanta, United States of America) was added. Test plates were further incubated for five days in a moist 35°C, 5% CO_2_ incubator to allow unbound antigen (either poliovirus serotype 1, serotype-2 or serotype-3) to infect the cells, with total cell death and detachment at the end of the five day incubation period. After the five-day incubation, the contents of the test plates were removed and replaced with a 0.05% crystal violet staining solution and allowed to stand at room temperature for 40 minutes. Crystal violet binds to proteins and DNA of adherent cells [29], thereby indicating the absence of unbound antigen and presence of specific antibody. Wells of test plates were then washed twice with ordinary tap water to remove unbound stain and allowed to air-dry. Plates were read using an ELISA plate reader at a wavelength of 570 nanometres. Optical density (OD) readings were transferred to an excel template from which poliovirus antibody titres were calculated. OD values <0.1 indicated virus infection (absence of neutralization and complete cell death and detachment) whereas OD values ≥0.1 indicated an intact cell monolayer and presence of specific antibody levels in the patient’s serum capable of total neutralisation of antigen. OD values <0.1 were assigned a value of “0” and OD values ≥0.1 were assigned a value of “1” for application of the Spearman-Kärber formula to obtain antibody titres [30]. Serum antibody neutralisation titres ranged from 5.66 (0/24 stained wells) to 1448.15 (24/24 stained wells). Each run included internal quality controls, cell controls and virus dilution controls for validation purposes. Polio neutralising antibody titres were determined for all three serotypes, as immunity to one does not protect an individual against another [31]. Serum antibody titres are expressed as the reciprocal of the highest dilution of serum which protects 50% of the cultures. A seropositive and sero-protective titre was defined as a neutralisation antibody titre of ≥8 (i.e. ≥3 log_2_) [32–37].

### Statistical analysis

We compared geometric mean antibody titres (GMTs) and percentage of infants with sero-protective titres at 6, 10 and 18 weeks of age, seroconversion rates at 10 and 18 weeks of age, and median fold change in antibody titre at 18 weeks of age versus 6 weeks of age in HEU, HIV+/ART+ and HIV+/ART- groups to HU infants as the referent group. Data was analysed using GraphPad Prism, Version 7.04 (GraphPad Software Inc, California, USA). GMTs and 95% confidence intervals (CIs) were determined following log_2_ transformation of serum antibody neutralisation titres as obtained following laboratory testing. Seroconversion rates at 10 weeks of age were based on a seronegative antibody titre at 6 weeks of age followed by a seropositive antibody titre at 10 weeks of age. Seroconversion rates at 18 weeks of age were based on a seronegative antibody titre at 6 and 10 weeks of age followed by a seropositive antibody titre at 18 weeks of age. Additionally, we compared GMTs, percentage of infants with sero-protective titres, seroconversion rates and median fold change in antibody titre between the HIV+/ART+ and HIV+/ART- groups. For comparisons of GMTs and fold change, the Kruskal-Wallis and Dunn’s multiple comparison post hoc test (one-way ANOVA of more than two groups) was used to compare HEU, HIV+/ART+ and HIV+/ART- groups to the HU group; whilst the Mann-Whitney test was used to compare differences between HIV+/ART+ and HIV+/ART- groups (head-to-head comparison of two groups). These tests accommodate differences in sample size between the groups. The Fisher Exact test was used to compare the percentage of infants with sero-protective titres and seroconversion rates between the groups, with application of the Newcombe/Wilson method to compute 95% CIs. P values of 0.05 or less were considered statistically significant.

### Ethics

Approval was obtained from the Faculty of Health Sciences, Higher Degrees Committee (HDC01-13-2014) and Academic Ethics Committee (AEC01-16-2014) of the University of Johannesburg, Johannesburg, South Africa. The initial parent study was also approved by the Human Research Ethics Committee at the University of the Witwatersrand (HREC: M080966) and registered at ClinicalTrials.gov (NCT00099658).

## Results

Demographic data as available and number of samples tested per group at each timepoint is shown in Table 1.

**Table 1:** Demographical data at 6, 10 and 18 weeks of age.

		HU^a^	HEU^b^	HIV+/ART+^c^	HIV+/ART-^d^
**Age 6 weeks**		*n* = 107	*n* = 116	*n* = 168	*n* = 74
	*Gender*				
	Male, Number (%)	56 (52.3)	62 (53.4)	65 (38.7)	27 (36.5)
	*Race*				
	Black, Number (%)	79 (73.8%)	108 (93.1%)	161 (95.8)	73 (98.6%)
	*Age in days (±SD)*				
	At last Vaccination	NA^e^	NA^e^	NA^e^	NA^e^
	At Sampling	49.4 (7.5)	51.5 (6.9)	52.8 (8.8)	51.4 (8.8)
	*Infants initiated on ART (%)*	-	-	100%	0%
**Age 10 weeks**		*n* = 107	*n* = 116	*n* = 166	*n* = 74
	*Gender*				
	Male, Number (%)	56 (52.3)	62 (53.4)	65 (39.2)	27 (36.5)
	*Race*				
	Black, Number (%)	79 (73.8%)	108 (93.1%)	159 (95.8%)	73 (98.6%)
	*Age in days (±SD)*				
	At last Vaccination	49.4 (7.5)	51.5 (6.9)	52.8 (8.8)	51.4 (8.8)
	At Sampling	77.9 (8.0)	80.2 (7.1)	80.5 (9.0)	79.9 (9.2)
	*Infants initiated on ART (%)*	-	-	100%	5.9%^f^
**Age 18 weeks**		*n* = 104	*n* = 114	*n* = 162	*n* = 70
	*Gender*				
	Male, Number (%)	55 (52.9)	60 (52.6)	61 (37.7)	26 (37.1)
	*Race*				
	Black, Number (%)	76 (73.1%)	106 (93.0%)	155 (95.7%)	69 (98.6%)
	*Age in days (±SD)*				
	At last Vaccination	106.3 (8.6)	108.5 (7.2)	108.9 (9.2)	108.4 (9.4)
	At Sampling	134.5 (8.9)	136.4 (7.1)	136.4 (9.1)	136.4 (10.8)
	*Infants initiated on ART (%)*	-	-	100%	17.9%^f^

^**a**^HIV-unexposed children as the referent group.

^**b**^HIV-exposed uninfected children.

^**c**^HIV-infected children on early ART initiated immediately at 6 weeks of age.

^**d**^HIV-infected children with deferred ART until clinically or immunologically indicated as per defined criteria.

^**e**^Vaccination age at birth not available.

^f^Estimated percentage of HIV+/ART- children initiated on ART at each time point following evaluations every 4 weeks after enrolment and randomisation at 6 weeks of age.

### Polio vaccine responses in HIV-infected infants on early ART (HIV+/ART+)

At 6 weeks of age, GMTs were lower in HIV+/ART+ compared to HU infants for serotype-1 (4.2 vs 4.8, p = 0.009), serotype-2 (5.9 vs 7.0, p = 0.009), and serotype-3 (4.1 vs 4.7, p = 0.041); Table 2 and S1 Fig. Furthermore, a lower percentage of HIV+/ART+ infants compared to HU infants had sero-protective titres to serotype-1 (57.1% vs 82.2%, p<0.001) and serotype-3 (47.6% vs 64.5%, p = 0.007); albeit with a similar trend for serotype-2 (86.3% vs 96.5%, p = 0.075); Table 2.

**Table 2:** Geometric mean titres and percentage of infants with sero-protective titres at 6, 10 and 18 weeks of age.

Group	Polio Serotype	Age 6 weeks	p value^e^	Age 10 weeks	p value^f^	Age 18 weeks	p value^g^
**HU**^a^		*n* = 107		*n* = 107		*n* = 104	
	GMT (95% CI)						
	1	4.8 (4.5–5.2)	-	6.8 (6.2–7.4)	-	8.5 (8.0–9.1)	-
	2	7.0 (6.5–7.4)	-	8.8 (8.4–9.2)	-	8.8 (8.5–9.1)	-
	3	4.7 (4.2–5.2)	-	7.5 (6.9–8.2)	-	8.5 (8.2–8.9)	-
	Percentage of infants with sero-protective titres (95% CI)						
	1	82.2%	-	89.7%	-	96.2%	-
	2	93.5%	-	98.1%	-	100.0%	-
	3	64.5%	-	87.9%	-	98.1%	-
**HEU**^b^		*n* = 116		*n* = 116		*n* = 114	
	GMT (95% CI)						
	1	4.0 (3.7–4.4)	0.005	6.3 (5.8–7.0)	>0.999	8.5 (8.0–9.0)	>0.999
	2	7.3 (6.9–7.8)	0.883	8.7 (8.3–9.2)	>0.999	9.0 (8.8–9.3)	>0.999
	3	4.6 (4.1–5.1)	>0.999	7.1 (6.6–7.7)	0.227	8.1 (7.7–8.6)	0.851
	Percentage of infants with sero-protective titres (95% CI)						
	1	61.2% (0.1–0.4)	0.001	82.8% (-0.0–0.3)	0.175	96.5% (-0.3–0.4)	0.740
	2	97.4% (-0.1–0.5)	0.201	98.3% (-0.4–0.4)	>0.999	100.0% (-0.4–0.7)	0.480
	3	53.4% (-0.0–0.2)	0.104	88.8% (-0.2–0.2)	0.838	95.6% (-0.2–0.5)	0.723
**HIV+/ART+**^c^		*n* = 168		*n* = 166		*n* = 162	
	GMT (95% CI)						
	1	4.2 (3.9–4.5)	0.009	5.1 (4.7–5.6)	<0.001	8.2 (7.7–8.7)	>0.999
	2	5.9 (5.5–6.3)	0.009	6.5 (6.0–6.9)	<0.001	8.0 (7.6–8.4)	0.133
	3	4.1 (3.7–4.4)	0.041	5.5 (5.1–6.0)	<0.001	7.9 (7.4–8.3)	0.843
	Percentage of infants with sero-protective titres (95% CI)						
	1	57.1% (0.2–0.4)	<0.001	65.1% (0.2–0.4)	<0.001	95.1% (-0.2–0.3)	>0.999
	2	86.3% (0.0–0.4)	0.075	89.8% (0.2–0.6)	0.007	96.3% (0.1–0.7)	0.251
	3	47.6% (0.0–0.3)	0.007	71.1% (0.1–0.4)	0.001	95.1% (-0.1–0.5)	0.536
**HIV+/ART-**^d^		*n* = 74		*n* = 74		*n* = 70	
	GMT (95% CI)						
	1	4.6 (4.1–5.2)	>0.999	5.4 (4.7–6.1)	0.032	6.1 (5.3–7.1)	0.022
	2	6.3 (5.7–7.0)	0.925	7.0 (6.3–7.8)	0.007	6.3 (5.5–7.1)	<0.001
	3	4.6 (4.0–5.2)	>0.999	6.1 (5.3–7.0)	0.184	5.9 (5.2–6.7)	<0.001
	Percentage of infants with sero-protective titres (95% CI)						
	1	71.6% (-0.2–0.3)	0.103	71.6% (0.1–0.5)	0.003	74.3% (0.3–0.7)	<0.001
	2	87.8% (0.1–0.4)	0.586	89.2% (0.2–0.8)	0.017	78.6% (0.5–0.9)	<0.001
	3	60.8% (-0.1–0.2)	0.641	71.6% (0.1–0.5)	0.007	75.7% (0.4–0.8)	<0.001

^**a**^HIV-unexposed children as the referent group.

^**b**^HIV-exposed uninfected children.

^**c**^HIV-infected children on early ART initiated immediately at 6 weeks of age.

^**d**^HIV-infected children with deferred ART until clinically or immunologically indicated as per defined criteria.

^**e**^p value at 6 weeks of age versus HIV-unexposed as the referent group.

^**f**^p value at 10 weeks of age versus HIV-unexposed as the referent group.

^**g**^p value at 18 weeks of age versus HIV-unexposed as the referent group.

At 10 weeks of age, GMTs remained lower in HIV+/ART+ compared to HU infants for serotype-1 (5.1 vs 6.8, p<0.001), serotype-2 (6.5 vs 8.8, p<0.001) and serotype-3 (5.5 vs 7.5, p<0.001); Table 2 and S1 Fig. Similarly, the percentage of infants with sero-protective titres were lower in HIV+/ART+ infants compared to HU infants for serotype-1 (65.1% vs 89.7%, p<0.001), serotype-2 (89.8% vs 98.1%, p = 0.007) and serotype-3 (71.1% vs 87.9%, p = 0.001); Table 2. At this time point, the seroconversion rate in HIV+/ART+ infants was higher than HU infants for serotype-1 (20.5% vs 9.3%, p = 0.018), but comparable for serotype-2 (7.8% vs 4.7%, p = 0.454) and serotype-3 (301.% vs 24.3%, p = 0.334); Table 3. At 18 weeks of age, GMTs (≥7.9 for all serotypes) and the percentage of children with sero-protective titres (≥95.1% for all serotypes) were similar in HIV+/ART+ infants compared to HU children; Table 2 and S1 Fig. Seroconversion rates at 18 weeks of age in HIV+/ART+ infants were higher than HU infants for serotype-1 (21.6% vs 7.7%, p = 0.003) and serotype-3 (21.1% vs 8.7%, p = 0.010), but comparable for serotype 2 (4.9% vs 1.9%, p = 0.324); Table 3. There was no significant difference in the median fold change in antibody titre at 18 weeks versus 6 weeks of age between HIV+/ART+ and HU infants for serotype-1 (25.4 vs 20.2, p = 0.641), serotype-2 (3.2 vs 3.2, p>0.999) and serotype-3 (20.2 vs 10.1, p = 0.944); Table 4.

**Table 3:** Seroconversion rates by 10 and 18 weeks of age.

		Seroconverted by 10 weeks (95% CI)	p value^e^	Seroconverted by 18 weeks (95% CI)	p Value^f^
**Serotype- 1**	HU^a^	9.3%	-	7.7%	-
	HEU^b^	30.2% (0.2–0.5)	<0.001	8.8% (-0.2–0.3)	0.810
	HIV+/ART+^c^	20.5% (0.1–0.4)	0.018	21.6% (0.1–0.4)	0.003
	HIV+/ART-^d^	12.2 (-0.2–0.3)	0.624	11.4% (-0.10.4)	0.432
**Serotype-2**	HU^a^	4.7%	-	1.9%	-
	HEU^b^	2.6% (-0.2–0.4)	0.485	0.0% (-0.3–0.6)	0.226
	HIV+/ART+^c^	7.8% (-0.1–0.4)	0.454	4.9% (0.0–0.6)	0.324
	HIV+/ART-^d^	6.8% (-0.2–0.4)	0.743	4.3% (-0.1–0.6)	0.392
**Serotype- 3**	HU^a^	24.3%	-	8.7%	-
	HEU^b^	38.8% (0.0–0.3)	0.022	7.9% (-0.2–0.3)	>0.999
	HIV+/ART+^c^	30.1% (-0.1–0.2)	0.334	21.0% (0.1–0.4)	0.010
	HIV+/ART-^d^	18.9% (-0.1–0.2)	0.467	17.1% (-0.0–0.4)	0.102

^**a**^HIV-unexposed children as the referent group.

^**b**^HIV-exposed uninfected children.

^**c**^HIV-infected children on early ART initiated immediately at 6 weeks of age.

^**d**^HIV-infected children with deferred ART until clinically or immunologically indicated as per defined criteria.

^**e**^p value at 10 weeks of age versus HIV-unexposed as the referent group.

^**f**^p value at 18 weeks of age versus HIV-unexposed as the referent group.

**Table 4:** Median fold change in antibody titre at 18 weeks of age versus 6 weeks of age.

		Serotyope-1	p value^e^	Serotype-2	p value^f^	Serotype-3	p value^g^
**HU**^a^	*n* = 104	20.2 (3.2–50.8)	-	3.2 (0.8–9.6)	-	10.1 (2.0–48.2)	-
**HEU**^b^	*n* = 114	36.2 (8.0–102.0)	0.196	2.5 (1.0–10.1)	>0.999	6.4 (1.0–80.6)	>0.999
**HIV+/ART+**^c^	*n* = 162	25.4 (1.5–128.0)	0.641	3.2 (1.0–20.2)	>0.999	20.2 (1.6–102.0)	0.944
**HIV+/ART-**^d^	*n* = 70	3.3 (0.5–34.1)	0.048	0.8 (0.2–5.4)	0.003	1.3 (0.4–25.4)	0.008

^**a**^HIV-unexposed children as the referent group.

^**b**^HIV-exposed uninfected children.

^**c**^HIV-infected children on early ART initiated immediately at 6 weeks of age.

^**d**^HIV-infected children with deferred ART until clinically or immunologically indicated as per defined criteria.

^**e**^p value of serotype-1 versus HUU as referent group.

^f^p value of serotype-2 versus HUU as referent group.

^g^p value of serotype-3 versus HUU as referent group.

### Polio vaccine responses in HIV infected infants with deferred ART (HIV+ART-)

At 6 weeks of age, no significant difference in GMTs were seen between HIV+/ART- and HU infants for serotype-1 (4.6 vs 4.8, p>0.999), serotype-2 (6.3 vs 7.0, p = 0.925) and serotype-3 (4.6 vs 4.7, p>0.999); Table 2 and S1 Fig. Likewise, there was no significant difference in the percentage of children with sero-protective titres between HIV+/ART- and HU infants for serotype-1 (71.6% vs 82.2%, p = 0.103), serotype-2 (87.7% vs 93.5%, p = 0.586) and serotype-3 (60.8% vs 64.5%, p = 0.641); Table 2. At 10 weeks of age, the GMTs were, however, significantly lower in HIV+/ART- than HU infants for serotype-1 (5.4 vs 6.8, p = 0.032) and serotype-2 (7.0 vs 8.8, p = 0.007); with a similar trend observed for serotype-3 (6.1 vs 7.5; p = 0.184); Table 2 and S1 Fig. Furthermore, sero-protective titres were significantly less prevalent in HIV+/ART- infants compared to HU infants for serotype-1 (71.6% vs 89.7%, p = 0.003), serotype-2 (89.2% vs 98.1%, p = 0.017) and serotype-3 (71.6% vs 87.9%, p = 0.007); Table 2. Seroconversion rates at 10 weeks of age were not significantly different in HIV+/ART- infants compared to HU infants for serotype-1 (12.2% vs 9.3%, p = 0.624), serotype 2 (6.8% vs 4.7%, p = 0.743) and serotype-3 (18.9% vs 24.3%, p = 0.467); Table 3. On clinical and/or immunological evaluation at 10 weeks of age, 5.9% of HIV+/ART- infants met the criteria for ART initiation and were initiated on ART. On evaluation at 14 weeks of age, at the time of the 14-week OPV dose, a further 4.9% of HIV+/ART- infants were initiated on ART. By 18 weeks of age, 5.9% of HIV+/ART- infants were on ART for approximately 10 weeks and 4.9% were on ART for approximately 4 weeks.

At 18 weeks of age, GMTs were significantly lower in HIV+ART- compared to HU infants for serotype-1 (6.1 vs 8.5, p = 0.022), serotype-2 (6.3 vs 8.8, p<0.001) and serotype-3 (5.9 vs 8.5, p<0.001); Table 2 and S1 Fig. In addition, the percentage of HIV+ART- children with sero-protective rates remained significantly lower than HU infants for serotype-1 (74.3% vs 96.2%, p<0.001), serotype-2 (78.6% vs 100.0%, p<0.001) and serotype-3 (75.7% vs 98.1%, p<0.001); Table 2. Notably, the percentage of HIV+/ART- infants with sero-protective titres to serotype-2 was lower at 18 weeks of age at 78.6% compared to 10 weeks of age at 89.2%. Seroconversion rates at 18 weeks of age were not significantly different between HIV+/ART- and HU infants for serotype-1 (11.4% vs 7.7%, p = 0.432), serotype 2 (4.3% vs 1.9%, p = 0.392) and serotype-3 (17.1% vs 8.7%, p = 0.102); Table 3. The median fold change in antibody titre at 18 weeks versus 6 weeks of age was lower in HIV+/ART- infants compared to HU infants for serotype-1 (3.3 vs 20.2, p = 0.048), serotype-2 (0.8 vs 3.2, p = 0.003) and serotype-3 (51.3 vs 10.1, p = 0.008); Table 4.

### Polio vaccine responses in HIV infected infants on early ART versus deferred ART (HIV+ART+ vs HIV+ART-)

At 6 weeks of age, the GMTs were not significantly different between HIV+/ART+ and HIV+/ART- infants for serotype-1 (4.2 vs 4.6, p = 0.113), serotype 2 (5.9 vs 6.3, p = 0.163) and serotype-3 (4.1 vs 4.6, p = 0.093); Table 2 and S1 Fig. The percentage of infants with sero-protective titres were also similar at 6 weeks of age between the HIV+/ART+ and HIV+/ART- groups for serotype-2 (86.3% vs 87.8%, p = 0.839) and serotype-3 (47.6% vs 60.8%; p = 0.070), albeit lower for serotype-1 in HIV+/ART+ infants compared to HIV+/ART- infants (57.1% vs 71.6%; p = 0.044); Table 2.

At 10 weeks of age, no significant difference in GMT was observed in HIV+/ART+ infants compared to HIV+/ART- infants for serotype-1 (5.1 vs 5.4, p = 0.509) and serotype-2 (6.5 vs 7.0, p = 0.079), although lower for serotype-3 in HIV+/ART+ infants compared to HIV+/ART- infants (5.5 vs 6.1; p = 0.037); Table 2. The percentage of infants with sero-protective titres were comparable between HIV+/ART+ infants and HIV+/ART- infants for serotype-1 (65.1% vs 71.6%, p = 0.373), serotype-2 (89.8% vs 89.2%, p>0.999) and serotype-3 (71.1% vs 71.6%, p>0.999); Table 2. Seroconversion rates at 10 weeks of age were not significantly different between HIV+/ART+ and HIV+/ART- infants for serotype-1 (20.5% vs 12.2%, p = 0.150), serotype-2 (7.8% vs 6.8%, p>0.999) and serotype-3 (30.1% vs 18.9%, p = 0.083); Table 3. However, by 18 weeks of age, GMTs were higher in HIV+/ART+ compared to HIV+/ART- infants for serotype-1 (8.2 vs 6.1; p = 0.003), serotype-2 (8.0 vs 6.3; p = 0.003), and serotype-3 (7.9 vs 5.9; p<0.001); Table 2. Additionally, a higher percentage of HIV+/ART+ compared to HIV+/ART- infants had sero-protective titres for serotype-1 (95.1% vs 74.3%; p<0.001); serotype-2 (96.3% vs 78.6%; p<0.001) and serotype-3 (95.1% vs 75.7%; p<0.001); Table 2. Seroconversion rates at 18 weeks of age were not significantly different between HIV+/ART+ and HIV+/ART- infants for serotype-1 (21.6% vs 11.4%, p = 0.096), serotype-2 (4.9% vs 4.3%, p>0.999) and serotype-3 (21.0% vs 17.1%, p = 0.592); Table 3. The median fold change in antibody titre at 18 weeks of age versus 6 weeks of age was significantly higher in HIV+/ART+ infants than HIV+/ART- infants for serotype-1 (25.4 vs 3.3, p<0.001), serotype-2 (3.2 vs 0.8, p<0.001) and serotype-3 (20.2 vs 1.3, p<0.001); Table 4.

### Polio vaccine responses in HIV-exposed uninfected infants

At 6 weeks of age, the GMT for serotype-1 was significantly lower in HEU infants compared to HU infants (4.0 vs 4.8, p = 0.005), although comparable for serotype-2 (7.3 vs 7.0, p = 0.883) and serotype-3 (4.6 vs 4.7, p>0.999); Table 2 and S1 Fig. Similarly, the percentage of HEU infants with sero-protective titres was lower than HU infants for serotype-1 (61.2% vs 82.2%; p = 0.001), and comparable for serotype-2 (97.4% vs 93.5%, p = 0.201) and serotype-3 (53.4% vs 64.5%, p = 0.104); Table 2. At 10 and 18 weeks of age, there were no differences in GMTs or percentage with sero-protective titres between HEU and HU infants for the three serotypes, with a sero-protective rate of ≥95.6% at 18 weeks of age in both groups; Table 2. Seroconversion rates were seen to be higher at 10 weeks of age in HEU infants compared to HU infants for serotype-1 (30.2% vs 9.3%, p<0.001) and serotype 3 (38.8 vs 24.3, p = 0.022), albeit comparable for serotype-2 (2.6 vs 4.7, p = 0.485). There was no significant difference in seroconversion rates at 18 weeks of age between HEU infants and HU infants for serotype-1 (8.8% vs 7.7%, p = 0.810), serotype-2 (0.0% vs 1.9%, p = 0.226) and serotype-3 (7.9% vs 8.7%, p>0.999); Table 3.

## Discussion

To our knowledge, this is the first study in which the timing of ART initiation in HIV-infected infants on neutralising antibody immune responses to trivalent OPV was systematically evaluated. Trivalent OPV, containing all three serotypes, has since been replaced in April 2016 with bivalent OPV, containing serotype-1 and serotype-3, in a globally co-ordinated switch following the declaration of serotype-2 eradication in September 2015 [38, 39].

Our study results corroborate findings in relation to other vaccines in that early initiation of ART in HIV-infected infants was generally associated with similar immune responses following the full primary series of OPV compared to HU infants, and superior immune responses compared to HIV-infected infants in whom ART was deferred until clinically or immunologically indicated [13, 20, 40–42]. In contrast, delaying ART until there was clinical or immunological disease progression in HIV-infected children was associated with impaired immune responses, including lower percentage of children with sero-protective titres, compared to either HU or HIV+/ART+ infants following the complete primary series of OPV. Notably, the immune responses in HEU infants to OPV was generally similar to HU infants throughout the study.

At 6 weeks of age, following the birth OPV dose, HIV+/ART+ infants, who were yet to be initiated on ART, had mostly lower polio immunity compared to HU infants. At 10 weeks of age, having initiated ART at 6 weeks of age, polio immunity remained lower in HIV+/ART+ infants compared to HU infants. The reduced transfer of maternal antibodies from HIV-infected mothers, as demonstrated by de Moraes-Pinto *et al*, and substantiated by the lower sero-protective (i.e. seropositive) rates at 6 and 10 weeks of age may have largely contributed to the lower immunity seen in HIV+/ART+ infants at these time points [43, 44]. The higher seroconversion rates observed in HIV+/ART+ infants at 10 and 18 weeks of age is also likely due to the lower seropositive rates at 6 and 10 weeks of age respectively. At 18 weeks of age, one month post the primary series of four OPV doses and approximately three months on ART, the antibody response in HIV+/ART+ children was comparable to HU infants, indicating that early initiation of ART resulted in reconstitution of the immune responses of these children to OPV.

Surprisingly, unlike infants of the HIV+/ART+ group, polio immunity in infants of the HIV+/ART- group were not significantly different from HU infants at 6 weeks of age, noting that at this time point infants of the HIV+/ART+ group had not initiated ART by then. The reason for the differences in antibody titres at 6 weeks of age between HIV+/ART+ and HIV+/ART- infants compared to HU infants is unclear. At 10 weeks of age, both HIV+/ART+ and HIV+/ART- infants demonstrated mostly inferior neutralizing antibody responses compared to HU infants, suggestive of early deleterious effects of HIV-infection on immune responses to OPV, even in children of CD4^+^ >25%. Impaired immune responses were also described by Troy *et al* in 9-month old HIV-infected children following at least three OPV doses, with the likelihood of some infants having initiated ART by then [27]. At 18 weeks of age, HIV+/ART- infants demonstrated significantly lower immune responses compared to HU and HIV+/ART+ infants, highlighting the benefits of early ART. Adequate immunity to polio is essential to eliminate the risk of re-emergence of disease and/or prolonged circulation of polioviruses [45–47]. The sero-protection rates amongst HIV+/ART- infants at 18 weeks of age is likely to decline if ART is not initiated and maintained, as a result of continuous deterioration of immune responses in these individuals coupled with declining maternal antibodies. The lower sero-protection rate in HIV+/ART- infants for serotype-2 at 18 weeks of age versus 10 weeks of age may be attributed to these conditions. This implies that any importation of wild poliovirus or circulating vaccine-derived poliovirus would likely circulate in HIV+/ART- individuals and could pose a risk to the general population.

As immunological aberrations have been described in HEU infants, evaluating the immunogenicity of OPV in these infants was warranted [7, 9, 10]. HEU infants are also prone to reduced maternal antibody transfer, and the likely reasons underlying differences to HU infants observed at early ages [43, 44]. A study by Church *et al* reported similar immune responses to OPV between HEU and HU Zimbabwean infants at 6 months of age following OPV doses at 3, 4 and 5 months of age, although an ELISA assay was used versus the gold standard neutralisation assay; the latter being a better measure of OPV induced immunity [25, 48, 49]. Tejiokem *et al* reported high seroconversion rates (≥96.2%) in HEU infants of Cameroon and Central African Republic aged 18 to 36 months who received at least four OPV doses in the first year of life [14]. Reassuringly, our findings corroborate the findings of those studies, and indicate that HEU infants mount similar functional antibody responses to OPV compared to HU infants.

Limitations of our study include the non-availability of a birth sample from which one could determine the level of maternal immunity. Study inclusion criteria were restricted to HIV-infected infants with CD4^+^ >25%; and therefore the results may not be generalizable to perinatal HIV-infected children with lower CD4^+^ counts. Also, the study was conducted during the period when only OPV was used in South Africa, with the schedule subsequently changed in 2009 to a combination of OPV at birth and 6 weeks of age, and inactivated polio vaccine at 6, 10 and 14 weeks of age with a booster at 18 months [50]. Although the immunogenicity of the combined OPV-IPV schedule in HIV-infected and/or HEU requires further investigation, the majority of sub-Saharan African countries with a high prevalence of HIV are still using OPV vaccines in their primary infant immunization programs.

In conclusion, HIV-infected infants not initiated on early ART demonstrated attenuated immune responses to OPV following a four-dose primary series, and might require additional booster doses of polio vaccine. In contrast, HIV-infected infants with ART initiated as early as 6 weeks of age, and HEU children demonstrated similar immunogenicity compared to HIV-unexposed infants following the primary four-dose OPV schedule. These data provide an additional argument for early HIV diagnosis and initiation of ART where applicable, in line with the WHO global recommendation which was based on the CHER study results [17, 51, 52].

## Supporting information

S1 Fig Scatter plot of geometric mean titre and 95% CI of log_2_ transformed antibody titre to polio seroype-1, serotype-2 and serotype-3 at 6, 10 and 18 weeks of age. (PDF)

S1 Dataset Raw data of polio antibody titres to serotype-1, serotype-2 and serotype-3 at 6, 10 and 18 weeks of age. (PDF)
