# Supplementary material for: Effect of HIV-exposure and timing of anti-retroviral treatment on immunogenicity of trivalent live-attenuated polio vaccine in infants
Source: PLoS One. 2019 Apr 19;14(4):e0215079. doi: 10.1371/journal.pone.0215079 (PMC6474646; doi:10.1371/journal.pone.0215079)
Supplement: S1 Fig — (PDF) [file pone.0215079.s001.pdf]

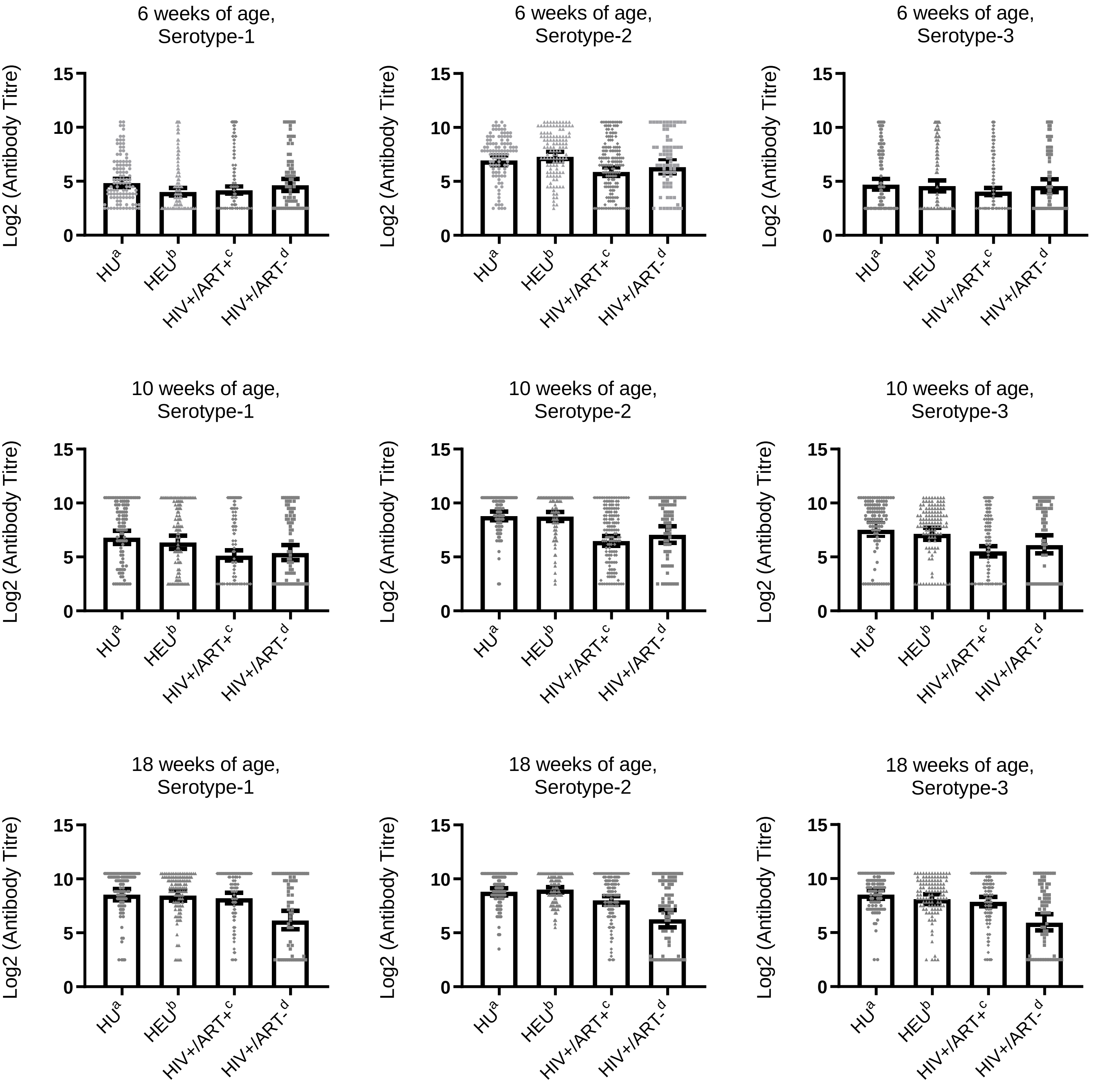

[a]HIV-unexposed children as the referent group. [b]HIV-exposed uninfected children, [c]HIV-infected children on early ART initiated immediately at 6 weeks of age, [d]HIV-infected children with deferred ART until clinically or immunologically indicated.

S1 Fig: Scatter plot of geometric mean titre and 95% CI of $\log_2$ transformed antibody titre to polio seroype-1, serotype-2 and serotype-3 at 6, 10 and 18 weeks of age.
