# Supplementary material for: Effect of HIV-exposure and timing of anti-retroviral treatment on immunogenicity of trivalent live-attenuated polio vaccine in infants
Source: PLoS One. 2019 Apr 19;14(4):e0215079. doi: 10.1371/journal.pone.0215079 (PMC6474646; doi:10.1371/journal.pone.0215079)
Supplement: S1 Dataset — (PDF) [file pone.0215079.s002.pdf]

HU, 6 weeks, Serotype 1	HEU, 6 weeks, Serotype 1	HIV+/ART-, 6 weeks, Serotype 1
5.66	35.92	1448.15
574.7	912.28	71.84
71.84	5.66	5.66
90.51	5.66	5.66
143.68	5.66	1448.15
5.66	22.63	181.02
912.28	5.66	574.7
114.04	7.13	574.7
8.98	17.96	1448.15
17.96	5.66	35.92
35.92	5.66	5.66
287.35	57.02	57.02
45.25	181.02	57.02
35.92	5.66	5.66
181.02	143.68	8.98
14.25	5.66	17.96
7.13	5.66	5.66
11.31	5.66	45.25
7.13	228.07	362.04
1448.15	7.13	35.92
57.02	14.25	114.04
28.51	22.63	8.98
11.31	11.31	5.66
5.66	8.98	14.25
28.51	11.31	5.66
71.84	11.31	1448.15
17.96	17.96	8.98
71.84	8.98	5.66
362.04	5.66	574.7
14.25	5.66	45.25
28.51	1149.4	28.51
11.31	5.66	45.25
11.31	11.31	90.51
362.04	5.66	22.63
11.31	28.51	5.66
35.92	228.07	5.66
11.31	5.66	5.66
1149.4	7.13	71.84
90.51	5.66	8.98
17.96	11.31	574.7
22.63	456.14	114.04
17.96	8.98	22.63
45.25	17.96	1448.15
35.92	11.31	181.02
456.14	22.63	1149.4
114.04	456.14	11.31
14.25	90.51	11.31
28.51	28.51	57.02
22.63	22.63	1448.15

90.51	5.66	5.66
71.84	45.25	912.28
181.02	7.13	8.98
28.51	22.63	8.98
456.14	5.66	5.66
8.98	5.66	45.25
1448.15	228.07	5.66
17.96	1448.15	28.51
11.31	14.25	5.66
22.63	724.08	5.66
14.25	11.31	5.66
114.04	5.66	22.63
22.63	7.13	7.13
5.66	5.66	362.04
71.84	8.98	22.63
574.7	1448.15	45.25
90.51	5.66	114.04
14.25	5.66	5.66
114.04	22.63	456.14
14.25	362.04	90.51
28.51	5.66	5.66
11.31	5.66	57.02
287.35	14.25	11.31
456.14	5.66	11.31
7.13	14.25	7.13
57.02	14.25	
5.66	912.28	
7.13	5.66	
22.63	11.31	
1149.4	71.84	
35.92	5.66	
17.96	71.84	
14.25	143.68	
7.13	1448.15	
228.07	287.35	
90.51	7.13	
114.04	22.63	
362.04	17.96	
57.02	22.63	
45.25	5.66	
14.25	5.66	
5.66	1448.15	
17.96	5.66	
5.66	28.51	
7.13	5.66	
5.66	45.25	
35.92	57.02	
5.66	5.66	
14.25	17.96	
5.66	57.02	

228.07	5.66	
5.66	5.66	
181.02	35.92	
5.66	724.08	
14.25	5.66	
7.13	45.25	
114.04	35.92	
57.02	5.66	
	14.25	
	7.13	
	181.02	
	7.13	
	45.25	
	22.63	
	8.98	
	114.04	
	14.25	

HIV+/ART+, 6 weeks, Serotype 1	HU, 6 weeks, Serotype 2	HEU, 6 weeks, Serotype 2
14.25	1149.4	45.25
5.66	71.84	574.7
11.31	287.35	1149.4
5.66	45.25	7.13
5.66	228.07	181.02
11.31	724.08	1448.15
574.7	724.08	724.08
724.08	287.35	574.7
7.13	912.28	228.07
724.08	912.28	1448.15
5.66	574.7	114.04
22.63	1149.4	1448.15
11.31	1149.4	724.08
7.13	912.28	143.68
22.63	574.7	287.35
5.66	574.7	8.98
5.66	287.35	90.51
22.63	57.02	456.14
5.66	7.13	35.92
5.66	228.07	1149.4
5.66	287.35	45.25
22.63	362.04	35.92
5.66	228.07	45.25
1448.15	114.04	181.02
181.02	287.35	90.51
11.31	45.25	1149.4
724.08	143.68	724.08
28.51	724.08	45.25
143.68	228.07	1149.4
5.66	912.28	71.84
5.66	181.02	143.68
912.28	7.13	143.68
5.66	362.04	287.35
5.66	181.02	287.35
22.63	362.04	1448.15
17.96	362.04	362.04
5.66	90.51	143.68
11.31	1149.4	574.7
574.7	71.84	456.14
57.02	228.07	574.7
28.51	143.68	287.35
7.13	35.92	1149.4
14.25	90.51	1149.4
35.92	362.04	57.02
28.51	5.66	574.7
5.66	724.08	574.7
28.51	90.51	456.14
5.66	181.02	362.04
1149.4	28.51	22.63

	7.13	287.35	22.63
	35.92	22.63	11.31
	7.13	114.04	1149.4
	22.63	912.28	14.25
	14.25	456.14	1448.15
	90.51	114.04	362.04
	5.66	574.7	912.28
	8.98	287.35	22.63
	5.66	362.04	57.02
	90.51	574.7	1448.15
	5.66	228.07	57.02
	22.63	1448.15	5.66
	362.04	724.08	228.07
	8.98	90.51	574.7
	71.84	228.07	1149.4
	574.7	574.7	456.14
	5.66	71.84	1149.4
	181.02	362.04	90.51
	143.68	574.7	1149.4
	5.66	17.96	228.07
	1448.15	14.25	456.14
	5.66	456.14	362.04
	5.66	11.31	287.35
	35.92	57.02	228.07
	22.63	57.02	574.7
	5.66	181.02	181.02
	1448.15	8.98	574.7
	5.66	45.25	181.02
	5.66	143.68	114.04
	5.66	22.63	1448.15
	5.66	228.07	143.68
	5.66	456.14	90.51
	5.66	45.25	1149.4
	1448.15	5.66	1448.15
	5.66	287.35	1448.15
	8.98	114.04	574.7
	5.66	456.14	22.63
	456.14	57.02	1149.4
	5.66	45.25	362.04
	45.25	228.07	7.13
	14.25	90.51	28.51
	5.66	90.51	143.68
	5.66	28.51	17.96
	90.51	362.04	143.68
	8.98	1448.15	724.08
	5.66	143.68	287.35
	14.25	7.13	912.28
	5.66	181.02	456.14
	5.66	228.07	14.25
	574.7	181.02	71.84

5.66	181.02	45.25
5.66	5.66	114.04
5.66	574.7	456.14
574.7	228.07	11.31
181.02	5.66	362.04
5.66	143.68	57.02
90.51	228.07	114.04
7.13	228.07	22.63
1448.15		143.68
45.25		143.68
7.13		456.14
912.28		724.08
5.66		57.02
5.66		287.35
574.7		90.51
5.66		57.02
5.66		22.63
1448.15		
71.84		
7.13		
5.66		
57.02		
17.96		
1149.4		
28.51		
90.51		
724.08		
71.84		
1448.15		
71.84		
5.66		
287.35		
1149.4		
228.07		
1448.15		
22.63		
5.66		
5.66		
5.66		
22.63		
11.31		
5.66		
7.13		
22.63		
90.51		
5.66		
22.63		
22.63		
5.66		
11.31		

11.31		
45.25		
912.28		
1448.15		
228.07		
181.02		
456.14		
11.31		
1448.15		
574.7		
5.66		
5.66		
5.66		
22.63		
5.66		
5.66		
5.66		
5.66		
5.66		

HIV+/ART-, 6 weeks, Serotype 2	HIV+/ART+, 6 weeks, Serotype 2	HU, 6 weeks, Serotype 3
1448.15	8.98	5.66
71.84	287.35	28.51
5.66	8.98	1448.15
1149.4	22.63	228.07
1149.4	45.25	90.51
1448.15	35.92	7.13
287.35	181.02	14.25
71.84	90.51	228.07
1448.15	1149.4	90.51
22.63	1149.4	1448.15
912.28	287.35	912.28
287.35	90.51	1149.4
143.68	574.7	456.14
1149.4	35.92	456.14
228.07	57.02	14.25
1448.15	90.51	5.66
1448.15	90.51	8.98
143.68	1448.15	362.04
114.04	287.35	5.66
90.51	5.66	71.84
1448.15	5.66	1149.4
181.02	22.63	22.63
28.51	362.04	22.63
11.31	90.51	5.66
181.02	1448.15	1448.15
912.28	45.25	228.07
7.13	724.08	1448.15
90.51	45.25	143.68
1149.4	71.84	181.02
71.84	5.66	181.02
45.25	5.66	574.7
22.63	1448.15	5.66
287.35	22.63	574.7
181.02	5.66	5.66
28.51	57.02	5.66
57.02	28.51	7.13
287.35	11.31	7.13
90.51	14.25	22.63
5.66	90.51	22.63
287.35	228.07	228.07
287.35	1149.4	71.84
456.14	45.25	22.63
456.14	57.02	11.31
35.92	5.66	5.66
287.35	228.07	5.66
228.07	228.07	181.02
28.51	143.68	362.04
57.02	11.31	71.84
5.66	5.66	5.66

5.66	22.63	362.04
57.02	143.68	114.04
11.31	724.08	5.66
5.66	57.02	181.02
5.66	143.68	5.66
1448.15	22.63	724.08
90.51	143.68	11.31
11.31	28.51	362.04
57.02	90.51	22.63
5.66	287.35	5.66
1448.15	7.13	5.66
11.31	8.98	17.96
90.51	71.84	11.31
5.66	5.66	17.96
1448.15	912.28	1149.4
287.35	228.07	14.25
1448.15	456.14	362.04
574.7	90.51	181.02
90.51	5.66	5.66
228.07	7.13	5.66
181.02	1149.4	5.66
1448.15	57.02	114.04
22.63	5.66	5.66
90.51	1448.15	287.35
45.25	228.07	11.31
	574.7	287.35
	5.66	5.66
	5.66	1448.15
	228.07	5.66
	287.35	5.66
	45.25	456.14
	228.07	143.68
	5.66	45.25
	90.51	5.66
	1149.4	5.66
	17.96	28.51
	5.66	5.66
	90.51	143.68
	5.66	11.31
	143.68	287.35
	114.04	5.66
	57.02	5.66
	5.66	11.31
	11.31	5.66
	143.68	228.07
	574.7	362.04
	22.63	8.98
	11.31	1149.4
	456.14	5.66
	228.07	5.66

		5.66	912.28
		1448.15	5.66
		90.51	5.66
		181.02	5.66
		287.35	7.13
		287.35	1448.15
		1448.15	7.13
		8.98	7.13
		287.35	
		22.63	
		14.25	
		724.08	
		287.35	
		90.51	
		114.04	
		28.51	
		71.84	
		456.14	
		11.31	
		5.66	
		11.31	
		912.28	
		114.04	
		114.04	
		143.68	
		1149.4	
		181.02	
		114.04	
		114.04	
		143.68	
		5.66	
		143.68	
		1448.15	
		724.08	
		143.68	
		228.07	
		574.7	
		57.02	
		57.02	
		28.51	
		28.51	
		17.96	
		28.51	
		143.68	
		181.02	
		1448.15	
		71.84	
		35.92	
		11.31	
		1448.15	

	17.96	
	1448.15	
	574.7	
	90.51	
	1448.15	
	362.04	
	724.08	
	57.02	
	143.68	
	57.02	
	1448.15	
	22.63	
	114.04	
	5.66	
	1149.4	
	45.25	
	912.28	
	114.04	
	143.68	

HEU, 6 weeks, Serotype 3	HIV+/ART-, 6 weeks, Serotype 3	HIV+/ART+, 6 weeks, Serotype 3
5.66	5.66	5.66
574.7	5.66	5.66
5.66	5.66	7.13
8.98	1149.4	5.66
5.66	228.07	5.66
5.66	1448.15	912.28
574.7	912.28	5.66
5.66	7.13	5.66
181.02	17.96	35.92
1448.15	456.14	1149.4
5.66	7.13	228.07
181.02	35.92	574.7
5.66	456.14	228.07
90.51	287.35	28.51
181.02	1448.15	57.02
5.66	574.7	456.14
5.66	5.66	5.66
5.66	5.66	5.66
90.51	228.07	5.66
5.66	287.35	5.66
90.51	14.25	5.66
724.08	45.25	5.66
22.63	5.66	11.31
5.66	181.02	5.66
287.35	5.66	11.31
912.28	228.07	14.25
5.66	22.63	5.66
1149.4	17.96	17.96
5.66	228.07	5.66
228.07	574.7	5.66
5.66	8.98	5.66
456.14	5.66	5.66
912.28	57.02	287.35
14.25	143.68	5.66
143.68	45.25	71.84
5.66	5.66	8.98
5.66	22.63	7.13
5.66	11.31	5.66
5.66	5.66	5.66
912.28	5.66	5.66
1448.15	1448.15	456.14
574.7	57.02	724.08
1448.15	5.66	7.13
5.66	5.66	5.66
1448.15	574.7	181.02
5.66	181.02	5.66
362.04	14.25	5.66
287.35	5.66	1149.4
456.14	5.66	5.66

1149.4	5.66	5.66
57.02	287.35	5.66
5.66	5.66	143.68
57.02	5.66	724.08
7.13	5.66	143.68
5.66	1149.4	5.66
11.31	22.63	11.31
5.66	5.66	8.98
5.66	14.25	228.07
5.66	5.66	5.66
362.04	5.66	5.66
5.66	574.7	71.84
7.13	912.28	5.66
5.66	11.31	5.66
5.66	181.02	5.66
143.68	287.35	5.66
228.07	5.66	287.35
90.51	5.66	5.66
912.28	5.66	14.25
5.66	114.04	5.66
8.98	181.02	71.84
5.66	1448.15	7.13
5.66	5.66	5.66
362.04	22.63	5.66
5.66	5.66	143.68
574.7		912.28
5.66		28.51
5.66		5.66
5.66		5.66
456.14		5.66
1149.4		8.98
5.66		5.66
574.7		7.13
143.68		181.02
456.14		1448.15
724.08		5.66
114.04		5.66
912.28		57.02
228.07		5.66
5.66		362.04
57.02		5.66
5.66		71.84
71.84		22.63
5.66		5.66
5.66		5.66
5.66		143.68
1448.15		574.7
5.66		5.66
7.13		5.66
5.66		5.66

1448.15		5.66
912.28		5.66
228.07		114.04
5.66		5.66
5.66		574.7
5.66		181.02
1448.15		574.7
574.7		5.66
5.66		5.66
5.66		1448.15
1448.15		5.66
57.02		5.66
8.98		5.66
14.25		5.66
574.7		90.51
5.66		90.51
5.66		5.66
		5.66
		45.25
		5.66
		5.66
		1448.15
		45.25
		5.66
		1448.15
		57.02
		5.66
		456.14
		114.04
		5.66
		5.66
		456.14
		574.7
		35.92
		181.02
		1149.4
		5.66
		28.51
		912.28
		287.35
		5.66
		5.66
		228.07
		228.07
		5.66
		5.66
		14.25
		7.13
		5.66
		5.66

		228.07
		287.35
		181.02
		1448.15
		456.14
		181.02
		5.66
		28.51
		181.02
		28.51
		35.92
		5.66
		5.66
		5.66
		912.28
		5.66
		362.04
		5.66
		228.07

HU, 10 weeks, Serotype 1	HEU, 10 weeks, Serotype 1	HIV+/ART-, 10 weeks, Serotype 1
28.51	45.25	1149.4
1448.15	724.08	181.02
1448.15	456.14	5.66
45.25	456.14	1448.15
287.35	1149.4	574.7
5.66	362.04	362.04
1448.15	5.66	456.14
456.14	912.28	287.35
114.04	7.13	362.04
1448.15	11.31	11.31
1448.15	912.28	5.66
143.68	28.51	1149.4
1448.15	22.63	90.51
574.7	45.25	45.25
1149.4	181.02	912.28
1149.4	5.66	7.13
5.66	5.66	5.66
912.28	181.02	11.31
5.66	574.7	456.14
912.28	22.63	11.31
1448.15	1448.15	22.63
362.04	1448.15	5.66
1448.15	1149.4	17.96
1448.15	724.08	5.66
181.02	912.28	5.66
574.7	8.98	1448.15
35.92	11.31	724.08
287.35	1448.15	5.66
1448.15	228.07	724.08
14.25	14.25	1448.15
1149.4	1149.4	5.66
5.66	45.25	22.63
1448.15	14.25	22.63
912.28	1448.15	11.31
1149.4	1448.15	5.66
35.92	456.14	724.08
5.66	1448.15	5.66
912.28	1448.15	45.25
90.51	5.66	5.66
71.84	8.98	143.68
14.25	724.08	1149.4
362.04	5.66	14.25
574.7	1448.15	1448.15
287.35	1149.4	35.92
456.14	114.04	1448.15
724.08	1149.4	5.66
8.98	1448.15	1448.15
22.63	22.63	11.31
14.25	45.25	1448.15

143.68	287.35	7.13
90.51	22.63	181.02
456.14	1448.15	5.66
1149.4	1448.15	5.66
574.7	1448.15	1448.15
1448.15	1448.15	362.04
1448.15	181.02	5.66
574.7	1448.15	456.14
11.31	228.07	1448.15
11.31	1448.15	5.66
14.25	1448.15	724.08
1149.4	362.04	912.28
912.28	228.07	5.66
724.08	45.25	90.51
724.08	14.25	1149.4
362.04	912.28	5.66
181.02	1448.15	28.51
17.96	1448.15	362.04
90.51	1448.15	287.35
22.63	8.98	228.07
90.51	5.66	5.66
1448.15	114.04	28.51
1448.15	5.66	574.7
1448.15	1448.15	14.25
8.98	228.07	287.35
181.02	90.51	
362.04	362.04	
57.02	5.66	
14.25	5.66	
228.07	114.04	
90.51	1448.15	
1448.15	57.02	
17.96	5.66	
5.66	71.84	
181.02	5.66	
228.07	574.7	
456.14	5.66	
1448.15	1448.15	
362.04	7.13	
228.07	228.07	
11.31	5.66	
5.66	362.04	
574.7	362.04	
7.13	724.08	
1149.4	5.66	
5.66	181.02	
1448.15	22.63	
1448.15	7.13	
912.28	7.13	
90.51	57.02	

181.02	1448.15	
5.66	724.08	
912.28	143.68	
228.07	1149.4	
181.02	1448.15	
5.66	35.92	
114.04	912.28	
45.25	1448.15	
	5.66	
	5.66	
	1149.4	
	228.07	
	45.25	
	11.31	
	1448.15	
	143.68	
	228.07	

HIV+/ART+, 10 weeks, Serotype 1	HU, 10 weeks, Serotype 2	HEU, 10 weeks, Serotype 2
724.08	1149.4	228.07
22.63	228.07	181.02
5.66	1149.4	1448.15
5.66	1448.15	90.51
228.07	1448.15	1448.15
	362.04	1149.4
362.04	1448.15	1448.15
287.35	181.02	1448.15
5.66	1448.15	287.35
71.84	228.07	1149.4
574.7	724.08	1448.15
1448.15	1448.15	574.7
456.14	912.28	574.7
	1149.4	912.28
5.66	1149.4	114.04
5.66	1448.15	71.84
5.66	143.68	287.35
14.25	1448.15	1448.15
1448.15	456.14	287.35
5.66	287.35	1448.15
5.66	1448.15	1149.4
5.66	1149.4	574.7
5.66	1448.15	90.51
456.14	724.08	1448.15
71.84	362.04	1448.15
5.66	456.14	574.7
228.07	143.68	1149.4
5.66	456.14	1149.4
8.98	287.35	1448.15
5.66	724.08	143.68
5.66	456.14	1448.15
362.04	5.66	574.7
1448.15	1448.15	181.02
5.66	1448.15	1448.15
5.66	912.28	1448.15
5.66	724.08	1448.15
912.28	114.04	724.08
5.66	1448.15	1448.15
181.02	456.14	287.35
7.13	287.35	362.04
8.98	90.51	1448.15
5.66	362.04	1149.4
5.66	1149.4	1448.15
17.96	1448.15	143.68
14.25	5.66	724.08
456.14	574.7	1448.15
5.66	90.51	724.08
574.7	1448.15	574.7
1448.15	287.35	90.51

181.02	912.28	90.51
17.96	1149.4	456.14
1448.15	228.07	1448.15
17.96	1448.15	1448.15
724.08	1448.15	1448.15
22.63	456.14	1448.15
5.66	1448.15	456.14
11.31	1448.15	724.08
5.66	1448.15	114.04
71.84	1448.15	1448.15
1149.4	456.14	35.92
1448.15	724.08	574.7
362.04	1448.15	1149.4
1448.15	90.51	1149.4
22.63	574.7	1448.15
181.02	362.04	228.07
5.66	1448.15	1448.15
45.25	362.04	1448.15
287.35	1149.4	1149.4
71.84	287.35	362.04
1448.15	1149.4	1149.4
1448.15	724.08	1149.4
1149.4	228.07	5.66
90.51	362.04	1448.15
5.66	724.08	1149.4
228.07	1149.4	362.04
1149.4	143.68	1448.15
1448.15	362.04	362.04
22.63	574.7	11.31
1448.15	114.04	456.14
456.14	912.28	362.04
5.66	456.14	1448.15
1149.4	45.25	362.04
1448.15	181.02	724.08
71.84	181.02	456.14
5.66	1448.15	7.13
5.66	1448.15	17.96
228.07	28.51	1448.15
45.25	912.28	287.35
362.04	228.07	228.07
7.13	90.51	35.92
724.08	574.7	1448.15
5.66	1149.4	1448.15
90.51	1448.15	1448.15
5.66	1448.15	456.14
5.66	90.51	574.7
7.13	1448.15	287.35
5.66	1448.15	1448.15
287.35	1448.15	228.07
574.7	228.07	143.68

5.66	181.02	724.08
362.04	143.68	1149.4
5.66	1448.15	1448.15
724.08	1448.15	1448.15
143.68	287.35	1448.15
5.66	362.04	57.02
1149.4	724.08	724.08
5.66	456.14	1448.15
724.08		181.02
45.25		1149.4
5.66		1448.15
287.35		1448.15
5.66		22.63
1448.15		574.7
228.07		287.35
90.51		456.14
5.66		90.51
574.7		
181.02		
5.66		
1448.15		
724.08		
8.98		
1448.15		
8.98		
28.51		
181.02		
724.08		
912.28		
143.68		
45.25		
1448.15		
1448.15		
1448.15		
1448.15		
8.98		
5.66		
7.13		
5.66		
228.07		
5.66		
5.66		
1448.15		
22.63		
22.63		
5.66		
912.28		
724.08		
456.14		
5.66		

90.51		
22.63		
724.08		
228.07		
362.04		
724.08		
287.35		
5.66		
574.7		
57.02		
90.51		
5.66		
5.66		
7.13		
1448.15		
5.66		
5.66		
5.66		
5.66		

HIV+/ART-, 10 weeks, Serotype 2	HIV+/ART+, 10 weeks, Serotype 2	HU, 10 weeks, Serotype 3
912.28	5.66	1149.4
45.25	362.04	724.08
5.66	456.14	1448.15
912.28	45.25	1448.15
287.35	57.02	362.04
1448.15		7.13
1448.15	90.51	1448.15
456.14	912.28	724.08
1448.15	1149.4	724.08
17.96	228.07	1149.4
287.35	1448.15	1149.4
912.28	1448.15	1448.15
71.84	1448.15	456.14
574.7		724.08
912.28	11.31	574.7
1448.15	35.92	143.68
1448.15	45.25	5.66
456.14	724.08	362.04
143.68	71.84	5.66
45.25	5.66	90.51
362.04	5.66	1448.15
1149.4	22.63	228.07
28.51	724.08	912.28
5.66	90.51	724.08
1448.15	724.08	912.28
1149.4	5.66	287.35
912.28	724.08	1448.15
17.96	8.98	362.04
1448.15	456.14	1448.15
71.84	5.66	1149.4
17.96	574.7	287.35
5.66	724.08	5.66
1448.15	1149.4	1149.4
35.92	5.66	456.14
362.04	11.31	5.66
90.51	28.51	574.7
228.07	362.04	5.66
45.25	5.66	912.28
5.66	8.98	912.28
114.04	114.04	1448.15
1448.15	362.04	228.07
181.02	11.31	181.02
1448.15	1448.15	912.28
17.96	5.66	724.08
574.7	181.02	574.7
287.35	90.51	90.51
228.07	22.63	456.14
90.51	574.7	574.7
456.14	22.63	724.08

1149.4	1448.15	456.14
71.84	228.07	57.02
5.66	574.7	5.66
5.66	912.28	1448.15
456.14	456.14	45.25
143.68	8.98	456.14
11.31	35.92	1448.15
362.04	35.92	228.07
362.04	181.02	362.04
5.66	912.28	1149.4
1448.15	181.02	228.07
574.7	14.25	362.04
1448.15	287.35	14.25
1149.4	45.25	181.02
1448.15	287.35	912.28
912.28	22.63	22.63
1448.15	1149.4	362.04
1448.15	90.51	574.7
912.28	5.66	90.51
181.02	1448.15	71.84
143.68	1448.15	287.35
574.7	1149.4	724.08
5.66	90.51	1448.15
724.08	1448.15	1448.15
17.96	1448.15	362.04
	456.14	574.7
	1448.15	228.07
	14.25	912.28
	456.14	287.35
	22.63	287.35
	181.02	1149.4
	14.25	362.04
	114.04	143.68
	71.84	5.66
	1448.15	5.66
	22.63	912.28
	7.13	181.02
	35.92	456.14
	114.04	574.7
	114.04	181.02
	228.07	5.66
	143.68	1448.15
	90.51	912.28
	5.66	1448.15
	114.04	287.35
	45.25	1448.15
	8.98	1149.4
	912.28	1448.15
	287.35	574.7
	181.02	114.04

		143.68	362.04
		912.28	228.07
		71.84	287.35
		724.08	287.35
		90.51	5.66
		143.68	1149.4
		574.7	5.66
		5.66	5.66
		362.04	
		1448.15	
		22.63	
		1448.15	
		287.35	
		724.08	
		287.35	
		724.08	
		35.92	
		362.04	
		90.51	
		5.66	
		45.25	
		287.35	
		456.14	
		287.35	
		1448.15	
		456.14	
		71.84	
		574.7	
		57.02	
		1448.15	
		8.98	
		90.51	
		1149.4	
		1448.15	
		1149.4	
		181.02	
		287.35	
		228.07	
		181.02	
		362.04	
		5.66	
		5.66	
		1448.15	
		912.28	
		228.07	
		1448.15	
		45.25	
		181.02	
		912.28	
		1149.4	

	7.13	
	1448.15	
	90.51	
	181.02	
	1448.15	
	1448.15	
	114.04	
	14.25	
	17.96	
	456.14	
	574.7	
	11.31	
	35.92	
	5.66	
	1149.4	
	11.31	
	724.08	
	11.31	
	114.04	

HEU, 10 weeks, Serotype 3	HIV+/ART-, 10 weeks, Serotype 3	HIV+/ART+, 10 weeks, Serotype 3
456.14	1448.15	5.66
228.07	912.28	5.66
724.08	5.66	228.07
114.04	1149.4	5.66
362.04	1149.4	456.14
456.14	1448.15	
1149.4	35.92	912.28
912.28	362.04	5.66
362.04	724.08	7.13
912.28	71.84	71.84
5.66	724.08	181.02
143.68	17.96	90.51
5.66	1448.15	114.04
456.14	71.84	
90.51	1149.4	5.66
362.04	1448.15	1149.4
5.66	574.7	5.66
228.07	287.35	362.04
28.51	181.02	228.07
8.98	456.14	28.51
57.02	574.7	5.66
287.35	287.35	5.66
5.66	5.66	912.28
574.7	35.92	181.02
57.02	1448.15	7.13
456.14	912.28	5.66
456.14	574.7	5.66
574.7	5.66	5.66
574.7	181.02	456.14
287.35	181.02	5.66
1448.15	5.66	5.66
912.28	5.66	912.28
362.04	724.08	724.08
287.35	5.66	5.66
287.35	724.08	22.63
724.08	5.66	1448.15
287.35	724.08	456.14
5.66	5.66	5.66
35.92	5.66	5.66
1149.4	1448.15	5.66
912.28	724.08	362.04
228.07	71.84	724.08
912.28	456.14	574.7
724.08	5.66	5.66
1448.15	724.08	71.84
1448.15	228.07	228.07
574.7	1149.4	287.35
287.35	5.66	1149.4
724.08	1448.15	143.68

228.07	5.66	8.98
57.02	57.02	574.7
181.02	5.66	71.84
1448.15	5.66	724.08
1448.15	5.66	181.02
5.66	1149.4	5.66
228.07	5.66	5.66
574.7	1448.15	5.66
45.25	1448.15	456.14
1149.4	5.66	362.04
228.07	912.28	362.04
362.04	1448.15	45.25
228.07	1448.15	1149.4
143.68	1448.15	71.84
1149.4	1149.4	11.31
114.04	90.51	5.66
143.68	1149.4	57.02
1448.15	5.66	5.66
912.28	287.35	5.66
143.68	90.51	574.7
5.66	362.04	1448.15
143.68	724.08	456.14
5.66	5.66	17.96
1149.4	5.66	1149.4
574.7	5.66	181.02
114.04		912.28
912.28		1448.15
456.14		5.66
11.31		114.04
456.14		114.04
912.28		1448.15
143.68		90.51
5.66		143.68
181.02		362.04
362.04		1149.4
362.04		90.51
57.02		5.66
1448.15		7.13
181.02		114.04
456.14		362.04
45.25		362.04
1149.4		287.35
1448.15		14.25
181.02		5.66
57.02		17.96
362.04		912.28
287.35		181.02
456.14		228.07
228.07		228.07
5.66		574.7

724.08		5.66
724.08		456.14
228.07		90.51
1149.4		912.28
287.35		181.02
5.66		11.31
724.08		362.04
90.51		5.66
5.66		287.35
287.35		724.08
724.08		5.66
456.14		1448.15
5.66		228.07
574.7		5.66
143.68		1448.15
28.51		90.51
228.07		5.66
		5.66
		14.25
		5.66
		228.07
		1448.15
		35.92
		5.66
		574.7
		1448.15
		5.66
		114.04
		57.02
		57.02
		17.96
		143.68
		456.14
		1448.15
		1149.4
		45.25
		287.35
		362.04
		456.14
		181.02
		90.51
		5.66
		71.84
		114.04
		362.04
		114.04
		228.07
		5.66
		362.04
		287.35

		45.25
		71.84
		724.08
		1448.15
		181.02
		1448.15
		5.66
		5.66
		17.96
		14.25
		912.28
		5.66
		5.66
		5.66
		287.35
		5.66
		1448.15
		5.66
		1149.4

HU, 18 weeks, Serotype 1	HEU, 18 weeks, Serotype 1	HIV+/ART-, 18 weeks, Serotype 1
287.35	912.28	17.96
724.08	362.04	5.66
1149.4	1448.15	
228.07	362.04	181.02
456.14	1149.4	114.04
724.08	724.08	45.25
362.04	574.7	1448.15
1448.15	362.04	1448.15
5.66	912.28	1448.15
1448.15	912.28	228.07
912.28	228.07	1448.15
181.02	1448.15	1448.15
1448.15	14.25	1448.15
456.14	456.14	1448.15
287.35	1448.15	1448.15
1149.4	1448.15	14.25
1149.4	574.7	5.66
1448.15	1149.4	1448.15
362.04	143.68	1448.15
456.14	1448.15	5.66
1448.15	1149.4	5.66
228.07	1149.4	143.68
1448.15	1448.15	5.66
1149.4	724.08	912.28
362.04	1149.4	1448.15
724.08	912.28	143.68
1448.15	362.04	5.66
456.14	362.04	1448.15
1448.15	574.7	362.04
1448.15	287.35	5.66
912.28	1149.4	7.13
181.02	90.51	1448.15
1448.15	1149.4	1448.15
1149.4	912.28	5.66
1448.15	574.7	574.7
22.63	1149.4	228.07
	1149.4	5.66
1149.4	456.14	912.28
574.7	57.02	1149.4
456.14	1448.15	1448.15
1149.4	574.7	14.25
912.28	1448.15	912.28
362.04	1149.4	5.66
1149.4	1448.15	1448.15
1149.4	228.07	912.28
1448.15	1448.15	362.04
1448.15	90.51	5.66
1448.15	228.07	228.07
1149.4	181.02	71.84

114.04	181.02	912.28
1448.15	456.14	5.66
1448.15	1448.15	912.28
1149.4	1448.15	724.08
912.28	1149.4	1149.4
1448.15	456.14	574.7
1149.4	912.28	114.04
1448.15	912.28	1448.15
1448.15	287.35	1448.15
5.66	1448.15	5.66
181.02	1448.15	57.02
362.04	1448.15	1448.15
912.28	724.08	11.31
1149.4	5.66	7.13
1448.15	724.08	574.7
90.51	574.7	456.14
22.63	724.08	45.25
45.25	1149.4	5.66
5.66	1149.4	5.66
287.35	181.02	45.25
456.14	90.51	5.66
574.7	1448.15	90.51
1149.4	574.7	
456.14	574.7	
1448.15	90.51	
1149.4	1149.4	
181.02	114.04	
287.35	912.28	
456.14	5.66	
143.68	71.84	
143.68	456.14	
1149.4	1149.4	
456.14	5.66	
143.68	1448.15	
1448.15	5.66	
228.07	1448.15	
1448.15	1149.4	
17.96	228.07	
1448.15	28.51	
1448.15	181.02	
114.04	574.7	
114.04	456.14	
1149.4	574.7	
90.51	456.14	
287.35	181.02	
912.28		
287.35	912.28	
287.35	1448.15	
912.28	228.07	
912.28	724.08	

181.02	14.25	
912.28	456.14	
456.14	71.84	
1149.4	71.84	
362.04	456.14	
5.66	1149.4	
	912.28	
	1448.15	
	143.68	
	287.35	
	114.04	
	912.28	
	912.28	
	724.08	
	143.68	
	912.28	

HIV+/ART+, 18 weeks, Serotype 1	HU, 18 weeks, Serotype 2	HEU, 18 weeks, Serotype 2
574.7	1149.4	362.04
1448.15	362.04	362.04
228.07	1448.15	1448.15
456.14	456.14	228.07
456.14	28.51	362.04
1448.15	724.08	912.28
90.51	1149.4	1149.4
912.28	228.07	574.7
1149.4	574.7	362.04
1448.15	574.7	574.7
1448.15	362.04	1149.4
724.08	1448.15	1149.4
456.14	456.14	1149.4
1448.15	287.35	181.02
228.07	181.02	114.04
90.51	28.51	362.04
1448.15	1448.15	1448.15
228.07	724.08	724.08
724.08	724.08	143.68
5.66	1448.15	912.28
1448.15	456.14	912.28
8.98	1149.4	181.02
5.66	1448.15	71.84
456.14	574.7	143.68
1448.15	114.04	1149.4
912.28	90.51	1448.15
1448.15	1448.15	456.14
1448.15	456.14	1448.15
1448.15	1448.15	1448.15
362.04	1149.4	181.02
17.96	1448.15	1448.15
181.02	287.35	181.02
1149.4	1448.15	1448.15
1149.4	574.7	724.08
5.66	1448.15	574.7
1448.15	143.68	724.08
1149.4		456.14
1448.15	1448.15	1448.15
1448.15	228.07	228.07
574.7	181.02	1448.15
1448.15	456.14	1448.15
1448.15	724.08	724.08
1448.15	1149.4	1149.4
724.08	1448.15	1448.15
22.63	456.14	1448.15
1448.15	362.04	1448.15
1149.4	1448.15	71.84
724.08	1448.15	143.68
1149.4	362.04	574.7

1448.15	456.14	114.04
5.66	362.04	228.07
574.7	912.28	912.28
1448.15	1448.15	181.02
1448.15	724.08	912.28
574.7	181.02	456.14
114.04	362.04	1149.4
724.08	724.08	228.07
45.25	1448.15	114.04
1448.15	90.51	1149.4
1448.15	90.51	724.08
456.14	456.14	724.08
114.04	456.14	724.08
1448.15	574.7	912.28
1149.4	574.7	1448.15
287.35	181.02	362.04
1448.15	287.35	456.14
724.08	724.08	1448.15
1448.15	362.04	287.35
1149.4	1448.15	574.7
1448.15	287.35	1149.4
1448.15	1149.4	1448.15
1448.15	114.04	1448.15
1448.15	362.04	912.28
1149.4	1448.15	228.07
1448.15	362.04	456.14
574.7	1448.15	724.08
574.7	362.04	362.04
143.68	724.08	143.68
1149.4	287.35	456.14
1448.15	1448.15	362.04
1448.15	1448.15	1149.4
574.7	45.25	57.02
912.28	287.35	181.02
912.28	143.68	228.07
724.08	90.51	1448.15
724.08	114.04	1448.15
1448.15	1448.15	1448.15
1448.15	1149.4	143.68
287.35	228.07	1448.15
1448.15	1149.4	362.04
574.7	1149.4	287.35
1448.15	143.68	912.28
574.7	143.68	1448.15
724.08	11.31	45.25
1448.15	1448.15	
5.66	1448.15	1448.15
1448.15	287.35	1149.4
1149.4	1448.15	456.14
456.14	1149.4	1448.15

287.35	912.28	181.02
5.66	1448.15	912.28
1149.4	362.04	1448.15
1448.15	456.14	143.68
1448.15	1448.15	456.14
1448.15	362.04	1448.15
1448.15		1448.15
228.07		1448.15
287.35		181.02
1448.15		362.04
1448.15		362.04
143.68		1149.4
1448.15		362.04
8.98		1448.15
71.84		181.02
287.35		1448.15
1149.4		
114.04		
1448.15		
287.35		
1149.4		
22.63		
28.51		
287.35		
1448.15		
1448.15		
45.25		
181.02		
1448.15		
724.08		
456.14		
287.35		
1448.15		
1448.15		
114.04		
724.08		
35.92		
1149.4		
287.35		
574.7		
28.51		
5.66		
456.14		
1448.15		
114.04		
8.98		
28.51		
11.31		
114.04		
574.7		

143.68		
228.07		
5.66		
228.07		
45.25		
90.51		
1149.4		
724.08		
71.84		
1448.15		
1149.4		
724.08		
1149.4		

HIV+/ART-, 18 weeks, Serotype 2	HIV+/ART+, 18 weeks, Serotype 2	HU, 18 weeks, Serotype 3
5.66	228.07	574.7
5.66	1149.4	912.28
	724.08	1448.15
90.51	7.13	574.7
143.68	1448.15	574.7
181.02	1448.15	287.35
228.07	228.07	912.28
1448.15	912.28	912.28
1448.15	1448.15	181.02
5.66	228.07	1149.4
287.35	362.04	228.07
1448.15	228.07	1149.4
181.02	181.02	362.04
912.28	1448.15	1448.15
724.08	912.28	143.68
228.07	1448.15	287.35
1448.15	724.08	143.68
1448.15	181.02	1448.15
181.02	22.63	1448.15
22.63	5.66	181.02
90.51	1448.15	724.08
114.04	1448.15	114.04
724.08	45.25	362.04
912.28	45.25	456.14
912.28	1448.15	362.04
114.04	11.31	362.04
5.66	1149.4	1448.15
724.08	362.04	143.68
1149.4	1448.15	1448.15
143.68	114.04	1448.15
7.13	181.02	1448.15
574.7	1448.15	5.66
181.02	1448.15	912.28
228.07	1149.4	1448.15
1448.15	5.66	228.07
22.63	1448.15	724.08
5.66	724.08	
7.13	35.92	1448.15
71.84	90.51	287.35
1448.15	1149.4	574.7
71.84	1448.15	143.68
574.7	1149.4	362.04
5.66	1149.4	287.35
1149.4	574.7	912.28
35.92	90.51	1448.15
5.66	1149.4	574.7
5.66	1149.4	912.28
362.04	1448.15	456.14
14.25	1448.15	574.7

1149.4	1448.15	362.04
5.66	228.07	1448.15
912.28	724.08	71.84
7.13	228.07	1448.15
1448.15	1149.4	228.07
362.04	912.28	143.68
5.66	456.14	181.02
1448.15	228.07	114.04
114.04	181.02	228.07
912.28	1448.15	143.68
362.04	1448.15	57.02
1448.15	143.68	456.14
287.35	228.07	724.08
143.68	1448.15	143.68
1149.4	1149.4	724.08
181.02	362.04	143.68
17.96	724.08	574.7
912.28	362.04	35.92
35.92	456.14	228.07
5.66	912.28	1448.15
35.92	724.08	228.07
5.66	912.28	1448.15
	912.28	1448.15
	1448.15	1448.15
	724.08	1448.15
	362.04	456.14
	1448.15	574.7
	456.14	181.02
	362.04	143.68
	1448.15	456.14
	574.7	912.28
	362.04	456.14
	362.04	912.28
	456.14	143.68
	287.35	912.28
	724.08	114.04
	574.7	1448.15
	181.02	287.35
	574.7	456.14
	5.66	912.28
	114.04	574.7
	1149.4	143.68
	90.51	57.02
	1149.4	1448.15
	143.68	724.08
	724.08	1149.4
	143.68	912.28
	228.07	724.08
	181.02	724.08
	228.07	1448.15

		1448.15	456.14
		45.25	114.04
		912.28	114.04
		1448.15	724.08
		574.7	574.7
		456.14	5.66
		1448.15	
		1149.4	
		228.07	
		1448.15	
		724.08	
		90.51	
		143.68	
		22.63	
		90.51	
		114.04	
		143.68	
		90.51	
		181.02	
		45.25	
		1149.4	
		57.02	
		5.66	
		71.84	
		1448.15	
		574.7	
		287.35	
		17.96	
		181.02	
		362.04	
		724.08	
		1448.15	
		362.04	
		724.08	
		181.02	
		90.51	
		143.68	
		228.07	
		362.04	
		574.7	
		57.02	
		114.04	
		28.51	
		181.02	
		45.25	
		362.04	
		912.28	
		362.04	
		22.63	
		912.28	

	181.02	
	912.28	
	5.66	
	8.98	
	362.04	
	724.08	
	456.14	
	181.02	
	912.28	
	1448.15	
	1448.15	
	456.14	
	456.14	

HEU, 18 weeks, Serotype 3	HIV+/ART-, 18 weeks, Serotype 3	HIV+/ART+, 18 weeks, Serotype 3
456.14	5.66	724.08
181.02	114.04	1448.15
287.35		181.02
574.7	912.28	114.04
114.04	45.25	724.08
362.04	5.66	90.51
287.35	143.68	574.7
114.04	57.02	362.04
912.28	14.25	362.04
1448.15	181.02	724.08
71.84	1448.15	71.84
71.84	456.14	362.04
912.28	1448.15	228.07
228.07	228.07	1448.15
1149.4	287.35	1448.15
1149.4	35.92	1448.15
1149.4	28.51	57.02
912.28	1448.15	228.07
5.66	287.35	90.51
724.08	724.08	45.25
574.7	114.04	287.35
574.7	35.92	1448.15
5.66	912.28	574.7
724.08	228.07	228.07
456.14	724.08	1448.15
228.07	287.35	5.66
90.51	5.66	181.02
181.02	456.14	362.04
1448.15	724.08	1448.15
456.14	362.04	1448.15
912.28	5.66	287.35
181.02	228.07	287.35
287.35	5.66	228.07
287.35	362.04	181.02
181.02	1149.4	5.66
912.28	114.04	724.08
362.04	7.13	1448.15
181.02	5.66	724.08
228.07	1448.15	114.04
1149.4	1448.15	1448.15
1448.15	45.25	912.28
1448.15	574.7	724.08
1448.15	5.66	724.08
456.14	1448.15	912.28
574.7	22.63	71.84
912.28	5.66	456.14
724.08	5.66	362.04
181.02	28.51	1448.15
724.08	7.13	574.7

114.04	1448.15	1448.15
143.68	5.66	114.04
724.08	287.35	71.84
287.35	5.66	1448.15
5.66	228.07	114.04
574.7	1448.15	574.7
1149.4	5.66	1448.15
28.51	1149.4	22.63
362.04	28.51	28.51
1448.15	181.02	456.14
912.28	5.66	912.28
456.14	724.08	456.14
912.28	181.02	143.68
114.04	574.7	1448.15
1448.15	912.28	114.04
1448.15	114.04	1448.15
574.7	17.96	1448.15
456.14	1448.15	143.68
724.08	143.68	1448.15
7.13	5.66	1448.15
362.04	362.04	1149.4
456.14	5.66	1448.15
1448.15		724.08
574.7		1149.4
181.02		228.07
456.14		574.7
362.04		724.08
114.04		1448.15
5.66		1448.15
143.68		181.02
1149.4		1448.15
1448.15		1448.15
57.02		5.66
287.35		90.51
71.84		143.68
1448.15		228.07
17.96		574.7
287.35		574.7
912.28		574.7
143.68		362.04
228.07		912.28
362.04		1448.15
1448.15		181.02
1149.4		912.28
1149.4		456.14
		1448.15
1448.15		5.66
1149.4		1448.15
143.68		57.02
456.14		1448.15

35.92		1448.15
181.02		5.66
228.07		1448.15
143.68		912.28
287.35		1448.15
228.07		1149.4
724.08		574.7
912.28		456.14
456.14		114.04
1149.4		143.68
724.08		362.04
287.35		28.51
724.08		724.08
287.35		28.51
143.68		181.02
574.7		90.51
		912.28
		287.35
		1448.15
		5.66
		228.07
		362.04
		143.68
		114.04
		1448.15
		1448.15
		5.66
		17.96
		287.35
		912.28
		143.68
		287.35
		1448.15
		574.7
		28.51
		90.51
		456.14
		362.04
		1448.15
		71.84
		456.14
		912.28
		71.84
		724.08
		8.98
		228.07
		57.02
		1149.4
		724.08
		143.68

		181.02
		362.04
		57.02
		5.66
		1448.15
		1149.4
		1448.15
		1448.15
		114.04
		574.7
		287.35
		14.25
		17.96
